# Modulation of Cytokine and Cytokine Receptor/Antagonist by Treatment with Doxycycline and Tetracycline in Patients with Dengue Fever

**DOI:** 10.1155/2011/370872

**Published:** 2011-03-28

**Authors:** J. E. Z. Castro, I. Vado-Solis, C. Perez-Osorio, T. M. Fredeking

**Affiliations:** ^1^Centro de Investigaciones Regionales “Dr. Hideyo Noguchi”, Universidad Autónoma de Yucatan, 97000 Mérida, YUC, Mexico; ^2^Facultad de Medicina, Universidad Autónoma de Yucatan, 97000 Mérida, YUC, Mexico; ^3^Antibody Systems Inc., 1901 Norwood Drive, Hurst, TX 76054, USA

## Abstract

Dengue virus infection can lead to dengue fever (DF) or dengue hemorrhagic fever (DHF). Disease severity has been linked to an increase in various cytokine levels. In this study, we evaluated the effectiveness of doxycycline and tetracycline to modulate serum levels of IL-6, IL-1B, and TNF and cytokine receptor/receptor antagonist TNF-R1 and IL-1RA in patients with DF or DHF. Hospitalized patients were randomized to receive standard supportive care or supportive care combined with doxycycline or tetracycline therapy. Serum cytokine and cytokine receptor/antagonist levels were determined at the onset of therapy and after 3 and 7 days. Cytokine and cytokine receptor/antagonist levels were substantially elevated at day 0. IL-6, IL-1**β**, and TNF remained at or above day 0 levels throughout the study period in untreated patients. Treatment with tetracycline or doxycycline resulted in a significant decline in cytokine levels. Similarly, IL-1RA and TNF-R1 serum concentrations were elevated at baseline and showed a moderate increase among untreated patients. Both drugs resulted in a significant rise in IL-1Ra levels by day 3 in patients. In contrast, treatment did not affect a similar result for TNF-R1. When compared to the control group, however, a significant rise post-treatment was seen upon intragroup analysis. Further analysis demonstrated that doxycycline was significantly more effective at modulating cytokine and cytokine receptor/antagonist levels than tetracycline.

## 1. Introduction

Dengue fever (DF) is a mosquito borne disease prevalent in tropical and subtropical areas of the world caused by 4 serotypes of Flavivirus [1]. The World Health Organization estimates 50 million cases occur annually in 100 countries with most patients presenting with flu-like symptoms [2]. Approximately 2.5 percent of those infected develop dengue hemorrhagic fever (DHF) characterized by prolonged very high fever, bleeding at mucosal surfaces, and significant hematological abnormalities, which can lead to circulatory collapse and death in 2.5–20% of cases [1–3]. Unfortunately, the last 2 decades have seen a marked increase in geographical distribution and significant outbreaks of both DF and DHF [2]. There is no vaccine against, or specific treatment for, either disease entity other than general supportive care.

What determines if infection leads to DF or DHF is not well understood, but prior exposure to dengue virus may play a critical role [2]. Cytokines are known to play a role in several viral hemorrhagic fevers including dengue [4, 5]. Previous studies have shown a correlation between increased levels of several cytokines and disease severity which may have prognostic value [5–8]. In general, these studies have shown that levels of cytokines adversely affecting coagulation tended to be higher in DHF versus DF [9, 10].

Given the critical role of cytokines in the inflammatory process and coagulopathies, there have been numerous attempts to suppress their levels in an attempt to control various diseases [11–13]. Various classes of antibiotics have been shown to possess immunomodulating properties [14]. Of these, drugs belonging to the tetracycline family were found to benefit patients with multiple sclerosis, Huntington's disease and rheumatoid arthritis presumably by suppressing microglia activity [14–18]. This, in turn, lowered levels of several proinflammatory cytokines including tissue necrosis factor (TNF) and interleukin 1 beta (IL-1*β*) [14, 18]. Recently, the levels of several proinflammatory cytokines in patients with tick-borne encephalitis (TBE) treated with tetracycline were found to be significantly lower than in un-treated controls [5]. 

The present study was conducted to evaluate the ability of tetracycline and doxycycline to modulate cytokines and cytokine soluble receptors and receptor antagonists in patients with DF or DHF.

## 2. Materials and Methods

### 2.1. Subjects

The study protocol was approved by the Independent University of Yucatan, Merida, Yucatan, Mexico. Informed consent was obtained from all adult patients and from the parents of children. Patients (34 untreated controls, 45 which received Doxycycline, and 35 which received tetracycline, see below) were recruited between June and December 2009 from hospitals in rural Latin America.

Blood samples were obtained from hospitalized patients 8–55 years of age presenting with symptoms characteristic of DF or DHF. For a presumptive diagnosis of DF or DHF (Day 0), a fever for more than 2 days accompanied by two or more of the following were present severe headache, retro-orbital pain, myalgia, arthralgia, rash, leucopenia, and hemorrhage. Serum was collected, and dengue virus infection was confirmed by PCR testing. DHF was defined as being PCR-positive for dengue virus accompanied by fever with one or more of the following being present positive tourniquet test for petechiae, mucosal hemorrhage, thrombocytopenia, an increase of >20% in hematocrit, or clinical evidence of shock. Patients were randomized within 72 hours of a confirmed diagnosis. All patients received symptomatic and supportive care as required. Doxycycline (Vibramycin, Pfizer, N.Y., USA) was administered as follows. For patients 18–55 years of age, an initial oral dose of 200 mg was followed by 100 mg administered at 12 hour intervals for 10 days. The same dosing regimen was used for patients 15–17 years of age, but only for 7 days. For those 8–14 years old, a single day 1 loading dose of 4 mg/kg/day was followed by 4/mg/kg/day divided between two doses given 12 hours apart for 7 days. Tetracycline (Tetrex, Hormona SA de C.V., Edo de Mexico, Mexico) was given as follows. In patients 18–55 years of age, 500 mg were administered orally at 8 hour intervals for 10 days. For those 15–17 years old, 500 mg were administered at 12 hour intervals for 7 days. Patients 8–14 years of age received 50 mg/kg/day divided between 3 doses given 8 hours apart for 7 days.

### 2.2. Diagnostic Tests

Infection with dengue virus was confirmed by PCR using multiple primers.

### 2.3. Cytokine Assays

Blood samples were processed to obtain serum which was stored at −70°C. Assays were performed in a blinded manner after all samples were collected. Serum cytokine and cytokine receptor levels were quantified using commercial ELISA tests kits (R & D Systems, Minneapolis, MN, USA) per the manufacturer's instructions. The limits of detection are as follows: IL (interleukin)-6, 0.7 pg/mL; IL-1*β*, <1 pg/mL; IL-1 receptor antagonist) (IL-1RA), 14 pg/mL; TNF, 0.6 pg/mL; and soluble TNF receptor (sTNF-R1), 3 pg/mL.

### 2.4. Statistical Analysis

Statistical analysis comparing cytokine and cytokine receptor/antagonist levels between controls (untreated) and treatment groups (intergroup analysis) was performed using the unpaired Student *t*-test. Data was adjusted for day zero values for each patient. Intragroup analysis (comparing day 0 versus day 3 and day 7 within the same group) was done using a paired Students *t*-test. A* P *value <  .05 was considered statistically significant.

## 3. Results

### 3.1. Effect of Doxycycline and Tetracycline Treatment on Serum Cytokine and Cytokine Receptor/Antagonist Levels in Patients with DF

All patients demonstrated elevated cytokine and cytokine receptor/antagonist levels at day 0 evidenced by higher mean levels in all study groups (Table 1). Over the 7-day observation period, proinflammatory cytokine (IL-6, IL-1*β*, and TNF) remained elevated in the control group. Similarly, IL1-RA and sTNF-R1 concentrations also remained elevated over the observation period. Treatment with doxycycline resulted in statistically (*P* < .01) lower levels of proinflammatory cytokines by day 3, both when compared to that seen at day 0 (intragroup analysis) and when compared to day 0 values in the control group. This decline continued with day 7 values significantly (*P* < .01) lower than those seen on day 3 (both inter- and intragroup). Treatment with tetracycline resulted in a similar trend, but generally it was not as rapid or pronounced. IL1-RA and TNF-R1 (molecules which downregulate cytokine activities) were found to be well above that seen in healthy individuals at time 0 in all patients. Increases between day 0 and day 7 values were modest in the control group. Treatment with doxycycline or tetracycline resulted in IL1-RA levels being significantly higher by day 3 and day 7, respectively. TNF-R1 levels were not significantly different between untreated patients and those receiving either tetracycline or doxycycline. Intragroup analysis comparing day 0 with day 3 and day 7 values demonstrated that both IL1-RA and TNF-R1 levels were significantly higher in all groups.

### 3.2. Effect of Doxycycline and Tetracycline Treatment on Serum Cytokine and Cytokine Receptor/Antagonist Levels in Patients with DHF

Doxycycline and tetracycline were also found to be effective at modulating cytokine and cytokine receptor/antagonist levels in patients with DHF (Table 2). Cytokine (IL-6, IL-1*β*, and TNF) levels were elevated in all groups at day 0 and remained so in untreated patients through day 7 (Table 2). Treatment with either doxycycline or tetracycline resulted in a significant (*P* < .01) reduction in cytokine levels by day 3 posttreatment when compared to either day 0 values in the control group or intragroup day 0 levels. This decline continued through day 7. Control patients with DHF also displayed a modest (15–30%) but significant (*P* < .01) rise in cytokine receptors/antagonists levels at day 3 and 7 compared to baseline (Table 2). Administration of doxycycline markedly enhanced this trend so that by day 3 and 7, IL-1RA and TNF-R1 levels had significantly (*P* < .01) increased when compared to day 0 levels in either the control or doxycycline treated groups. In contrast, the administration of tetracycline did not affect a significant (*P* > .05) rise in either IL1-RA or TNF-R1 serum concentrations at either day 3 or day 7 when compared to controls (intergroup analysis). However, intragroup analysis did show that tetracycline significantly (*P* < .05) increased TNF-R1 levels at both day 0 and 7. No similar effect was seen for IL-1RA.

### 3.3. Differential Effect of Doxycycline and Tetracycline on Serum Cytokine and Cytokine Receptor/Antagonist Levels

The above results indicated that both doxycycline and tetracycline were effective at modulating serum cytokine and cytokine receptor/antagonist response in patients with DF and DHF. However, in some instances, doxycycline appeared to be more effective. We, therefore, compared cytokine levels in patients with DF and DHF after 3 and 7 days of treatment (Table 3). Day 3 levels for proinflammatory cytokines IL-1*β* and TNF-*α* and were significantly lower in patients with DF or DHF who received doxycycline versus tetracycline. IL-6 levels at day 3 were comparable (*P* > .05) in patients treated with either drug. By day 7, IL-6 concentrations were significantly (*P* < .01) lower in the group receiving doxycycline versus tetracycline. In patients with DHF, doxycycline was significantly more effective at lowering IL-6 levels at both days 3 and 7. A similar effect was seen for cytokine receptor/antagonist levels. Therefore, administration of doxycycline significantly raised IL1-RA and TNF-R1 above those observed with tetracycline both at day 3 and day 7.

## 4. Discussion

Elevated cytokine levels are a hallmark of numerous bacterial and viral infectious diseases including dengue [5, 7–9]. Proinflammatory cytokines, such as IL-6, IL1-*β* and TNF, are believed to cause the majority of symptoms, such as fever, malaise, and coagulopathies associated with infections. Indeed, the degree of imbalance between such cytokines and their anti-inflammatory counterparts may be the primary prognostic indicator of disease outcome [19–21]. These finding have led to the development of a broad spectrum of potential therapeutic agents, including monoclonal antibodies and antibiotics, which act to downregulate various cytokines [22–25]. Drugs belonging to the tetracycline class of antibiotics possess several advantages including a long history of safe use and low cost. Additionally, their ability to cross the blood-brain barrier with relative ease may prove critical in the treatment of infections involving the central nervous system. Atrasheuskaya et al. [5] have shown that administration of tetracycline to patients with tick-borne encephalitis affected a marked positive shift in the ratio of cytokines to their respective soluble receptors. Such changes in cytokine to soluble receptor ratios appear to be more critical to disease resolution than the absolute level of cytokines themselves [26, 27]. 

In the present study, we investigate the effectiveness of tetracycline and doxycycline to modulate the levels of various cytokines and soluble receptor/receptor antagonists in patients with DF or DHF. As has been previously observed [6–9], dengue virus infection resulted in a marked increase in serum cytokine and cytokine receptor/antagonist levels. Both drugs were able to modulate proinflammatory cytokines levels. Downregulation was rapid, being observed within 3 days of treatment and continuing through day 7. A similar effect was noted for IL-1RA but not for TNF-R1. In patients with DF, TNF-R1 levels were not statistically different when control patients were compared to either treatment group. An intragroup analysis did achieve significance indicating that differences in baseline levels between groups at the time therapy was initiated may have a substantial influence. In the case of DHF, doxycycline showed a clear superiority to tetracycline in modulating TNF-R1 concentration. A direct comparison between tetracycline and doxycycline demonstrated that the later was a far more effective immunomodulator. This can most likely be attributed to the fact that doxycycline has a longer plasma half-life than tetracycline. Both drugs appeared to exert the same degree of effectiveness in patients with DF or DHF, a much more severe disease syndrome. 

An additional potential benefit to using doxycycline in the treatment of DF or DHF is its recently discovered ability to inhibit dengue virus multiplication in tissue culture [28]. Doxycycline, but not tetracycline, was able to interact with the dengue virus E protein to inhibit a conformational change which is an essential step in the process by which the virus enters susceptible cells.

The present study indicates that doxycycline may provide a clinical benefit in the treatment of dengue virus infection by modulating the cytokine cascade. Unfortunately, the study design did not allow for us to determine this. We hope to initiate a study in the near future to determine if doxycycline can provide a clinical benefit to patients with DF or DHF by monitoring disease severity, mortality rates, and time of hospital discharge postenrollment. 

## Figures and Tables

**Table 1:** Effect of Doxycycline and Tetracycline on cytokine levels and IL1-RA and S TNF-R1 in patients with dengue fever.

Treatment group (GMT and ranges, pg/mL)									

	Control			Doxycycline			Tetracycline		

	Day 0	Day 3	Day 7	Day 0	Day 3	Day 7	Day 0	Day 3	Day 7
IL-6	6.99	7.58	7.41	7.47	5.98*	3.96*	7.38	6.36*	5.48*
	(5.59–8.32)	(6.24–8.98)	(4.18–9.30)	(6.28–9.16)	(3.70–7.98)	(2.32–6.89)	(5.33–9.39)	(4.72–7.90)	(3.89–7.07)
IL-1*β*	4.53	4.95	4.64	4.53	2.29*	1.55*	4.41	3.36*	2.23*
	(3.32–6.30)	(3.67–6.54)	(2.25–6.43)	(3.35–6.36)	(1.23–4.12)	(1.10–2.78)	(3.15–6.27)	(1.48–5.43)	(1.02–4.69)
IL1 RA	325	344	349	264	673*	964*	263	330*	404*
	(189–632)	(202–714)	(199–699)	(187–381)	(486–851)	(701–1459)	(184–366)	(201–732)	(231–920)
TNF	4.38	5.02	5.20	4.46	2.42*	0.90*	4.37	3.89*	3.31*
	(2.69–6.43)	(2.85–7.89)	(3.10–8.21)	(2.66–6.54)	(0.23–4.63)	(0.4–3.02)	(2.67–6.22)	(2.03–5.65)	(1.19–5.08)
TNF R1	1751	2074	2189	1969	2432^†^	2616^†^	1816	2115^†^	2324^†^
	(989–2899)	(1143–3304)	(1200–3278)	(298–3312)	(673–3762)	(1021–4412)	(1099–2766)	(1358–3282)	(1409–3974)

*(*P* < .01).

^†^(*P* > .05).

**Table 2:** Effect of Doxycycline and Tetracycline on cytokine levels and IL1-RA and S TNF-R1 in patients with dengue hemorrhagic fever.

Treatment group (GMT and ranges, pg/mL)									

	Control			Doxycycline			Tetracycline		
	Day 0	Day 3	Day 7	Day 0	Day 3	Day 7	Day 0	Day 3	Day 7

IL-6	4.75	5.22	5.20	5.54	3.84*	2.36*	5.11	4.68*	4.23*
	(3.28–7.24)	(3.24–7.89)	(3.27–8.04)	(3.42–7.65)	(2.01–6.73)	(1.24–5.38)	(3.27–7.28)	(2.93–6.98)	(2.46–6.66)
IL-1*β*	6.88	7.83	8.07	7.56	5.32*	3.29*	7.97	7.68*	7.22*
	(3.32–9.32)	(4.61–10.43)	(3.25–10.56)	(4.84–10.30)	(3.21–8.53)	(1.21–6.48)	(6.01–9.81)	(5.59–9.44)	(5.21–9.32)
IL1 RA	494	611	676	335	710*	996*	497	517^†^	510^†^
	(314–671)	(307–812)	(455–981)	(221–472)	(462–928)	(703–1532)	(285–672)	(342–695)	(342–714)
TNF	7.76	8.44	8.50	6.97	5.31*	4.31*	8.05	7.83*	7.54*
	(6.11–10.20)	(6.72–11.28)	(6.02–11.10)	(1.12–11.01)	(0.46–12.32)	(1.04–7.94)	(6.28–9.73)	(5.99–9.62)	(5.71–9.17)
TNF R1	1593	1719	1796	1497	1961*	2367*	1631	1736^†^	1853^†^
	(1284–1902)	(1273–2135)	(1279–2387)	(1212–1843)	(1538–2985)	(1759–3129)	(1271–1961)	(1329–2116)	(1365–2362)

*(*P* < .01).

^†^(*P* > .05).

**Table 3:** Differential effect of Doxycycline and Tetracycline on cytokine levels and IL1-RA and S TNF-R1 in patients with dengue fever and dengue hemorrhagic fever.

Dengue fever (GMT and ranges, pg/mL)					DHF (GMT and ranges, pg/mL)			

	Doxycycline		Tetracycline		Doxycycline		Tetracycline	
	Day 3	Day 7	Day 3	Day 7	Day 3	Day 7	Day 3	Day 7

IL-6	5.98^†^	3.96*	6.36	5.48	3.84*	2.36*	4.68	4.23
	(3.70–7.98)	(2.32–6.89)	(4.72–7.90)	(3.89–7.07)	(2.01–6.73)	(1.24–5.38)	(2.93–6.98)	(2.46–6.66)
IL-1*β*	2.29*	1.55*	3.36	2.23	5.32*	3.29*	7.68	7.22
	(1.23–4.12)	(1.10–2.78)	(1.48–5.43)	(1.02–4.69)	(3.21–8.53)	(1.21–6.48)	(5.59–9.44)	(5.21–9.32)
IL-1 RA	673*	964*	330	404	710*	996*	517	510
	(486–851)	(701–1459)	(201–732)	(231–920)	(462–928)	(703–1532)	(342–695)	(342–714)
TNF	2.42*	0.90*	3.89	3.31	5.31*	4.31*	7.83	7.54
	(0.23–4.63)	(0.4–3.02)	(2.03–5.65)	(1.19–5.08)	(0.46–12.32)	(1.04–7.94)	(5.99–9.62)	(5.71–9.17)
TNF R1	2432*	2616^€^	2115	2324	1961*	2367*	1736	1853
	(673–3762)	(1021–4412)	(1358–3282)	(1409–3974)	(1538–2985)	(1759–3129)	(1329–2116)	(1365–2362)

^€^(*P* < .05).

*(*P* < .01).

^†^(*P* > .05).

## References

[1] Gubler DJ (2002). Epidemic dengue/dengue hemorrhagic fever as a public health, social and economic problem in the 21st century. *Trends in Microbiology*.

[2] Pang T, Cardosa MJ, Guzman MG (2007). Of cascades and perfect storms: the immunopathogenesis of dengue haemorrhagic fever-dengue shock syndrome (DHF/DSS). *Immunology and Cell Biology*.

[3] Guzman MG, Kouri G (2003). Dengue and dengue hemorrhagic fever in the Americas: lessons and challenges. *Journal of Clinical Virology*.

[4] Marty AM, Jahrling PB, Geisbert TW (2006). Viral hemorrhagic fevers. *Clinics in Laboratory Medicine*.

[5] Atrasheuskaya AV, Fredeking TM, Ignatyev GM (2003). Changes in immune parameters and their correction in human cases of tick-borne encephalitis. *Clinical and Experimental Immunology*.

[6] Bozza FA, Cruz OG, Zagne SMO (2008). Multiplex cytokine profile from dengue patients: MIP-1beta and IFN-gamma as predictive factors for severity. *BMC Infectious Diseases*.

[7] Bethell DB, Flobbe K, Phuong CXT (1998). Pathophysiologic and prognostic role of cytokines in dengue hemorrhagic fever. *Journal of Infectious Diseases*.

[8] Hober D, Poli L, Roblin B (1993). Serum levels of tumor necrosis factor-*α* (TNF-*α*), interleukin-6 (IL-6), and interleukin-1*β* (IL-1*β*) in dengue-infected patients. *American Journal of Tropical Medicine and Hygiene*.

[9] Green S, Vaughn DW, Kalayanarooj S (1999). Early immune activation in acute dengue illness is related to development of plasma leakage and disease severity. *Journal of Infectious Diseases*.

[10] Suharti C, van Gorp ECM, Setiati TE (2002). The role of cytokines in activation of coagulation and fibrinolysis in dengue shock syndrome. *Thrombosis and Haemostasis*.

[11] Feldmann M (2008). Many cytokines are very useful therapeutic targets in disease. *Journal of Clinical Investigation*.

[12] Phelan JD, Orekov T, Finkelman FD (2008). Cutting edge: mechanism of enhancement of in vivo cytokine effects by anti-cytokine monoclonal antibodies. *Journal of Immunology*.

[13] Ratsimandresy RA, Rappaport J, Zagury JF (2009). Anti-cytokine therapeutics: history and update. *Current Pharmaceutical Design*.

[14] Tauber SC, Nau R (2008). Immunomodulatory properties of antibiotics. *Current Molecular Pharmacology*.

[15] Bonelli RM, Hodl AK, Hofmann P, Kapfhammer HP (2004). Neuroprotection in Huntington’s disease: a 2-year study on minocycline. *International Clinical Psychopharmacology*.

[16] Zabad RK, Metz LM, Todoruk TR (2007). The clinical response to minocycline in multiple sclerosis is accompanied by beneficial immune changes: a pilot study. *Multiple Sclerosis*.

[17] Stone M, Fortin PR, Pacheco-Tena C, Inman RD (2003). Should tetracycline treatment be used more extensively for rheumatoid arthritis? Metaanalysis demonstrates clinical benefit with reduction in disease activity. *Journal of Rheumatology*.

[18] Lai AY, Todd KG (2006). Hypoxia-activated microglial mediators of neuronal survival are differentially regulated by tetracyclines. *GLIA*.

[19] Girardin E, Roux-Lombard P, Grau GE, Suter P, Gallati H, Dayer J-M (1992). Imbalance between tumour necrosis factor-alpha and soluble TNF receptor concentrations in severe meningococcaemia. *Immunology*.

[20] Lehmann AK, Halstensen A, Sornes S, Rokke O, Waage A (1995). High levels of interleukin 10 in serum are associated with fatality in meningococcal disease. *Infection and Immunity*.

[21] van Dissel JT, van Langevelde P, Westendorp RGJ, Kwappenberg K, Frölich M (1998). Anti-inflammatory cytokine profile and mortality in febrile patients. *Lancet*.

[22] Dinarello CA (2003). Anti-cytokine therapeutics and infections. *Vaccine*.

[23] Zheng BJ, Chan KW, Lin YP (2008). Delayed antiviral plus immunomodulator treatment still reduces mortality in mice infected by high inoculum of influenza A/H5N1 virus. *Proceedings of the National Academy of Sciences of the United States of America*.

[24] Panacek EA, Marshall JC, Albertson TE (2004). Efficacy and safety of the monoclonal anti-tumor necrosis factor antibody F(ab’)_2_ fragment afelimomab in patients with severe sepsis and elevated interleukin-6 levels. *Critical Care Medicine*.

[25] Clark IA, Budd AC, Alleva LM, Cowden WB (2006). Human malarial disease: a consequence of inflammatory cytokine release. *Malaria Journal*.

[26] Dinarello CA (1994). The biological properties of interleukin-1. *European Cytokine Network*.

[27] Pruitt JH, Welborn MB, Edwards PD (1996). Increased soluble interleukin-1 type II receptor concentrations in postoperative patients and in patients with sepsis syndrome. *Blood*.

[28] Yang JM, Chen YF, Tu YY, Yen KR, Yang YL (2007). Combinatorial computational approaches to identify tetracycline derivatives as flavivirus inhibitors. *PLoS ONE*.

